# “Holding the line”—relationship building and challenges when nursing adults with a severe eating disorder

**DOI:** 10.1186/s40337-024-01155-0

**Published:** 2024-12-02

**Authors:** Berit Støre Brinchmann, Kathrine Rasch Moyo, Kristin Anne Stavnes

**Affiliations:** 1 Regional Centre for Eating Disorders, Nordland Hospital Trust, Bodø, Norway; 2 Faculty of Nursing and Health Sciences, Nord University, Bodø, Norway

**Keywords:** Ambivalence, Anorexia nervosa, Anxiety, Challenge, Eating disorder, Nursing, Relationship, Resistance, Rigidity

## Abstract

**Background:**

Eating disorders (EDs) are serious psychiatric disorders that may cause great suffering and reduced quality of life. Severe EDs often lead to somatic complications and have a high mortality rate. The most seriously ill patients need hospitalisation, with a need for highly trained nurses.

**Methods:**

The aim of the study was to investigate challenges when nursing adults with a severe ED, and how to succeed in establishing a therapeutic relationship. A qualitative design was chosen, and individual qualitative interviews conducted with twelve nurses. The analytic method was Braun and Clarke's thematic analysis.

**Results:**

Six main themes were developed: entering the patient’s world of experience (with the subthemes: understanding the patient’s anxiety, understanding the patient's rigidity and need for control and understanding the patient's ambivalence), providing somatic nursing, building relationships, ‘holding the line’ (with the subtheme: demanding and rewarding), manoeuvring between rules and frameworks and providing good nursing care during coercive treatment.

**Conclusion:**

Nursing patients with a severe ED is demanding, requiring somatic as well as psychiatric expertise. The nurse must understand the patient's anxiety, rigidity and need for control, and ambivalence. Nursing patients with a severe ED requires clarity, and steadiness and the nurses must cope with resistance. To succeed, teamwork and support from colleagues are needed.

**Supplementary Information:**

The online version contains supplementary material available at 10.1186/s40337-024-01155-0.

## Abstract (plain language summary)

Eating disorders (EDs) are psychiatric disorders with potentially serious and life-threatening consequences, and it is important to develop knowledge about appropriate nursing for these patients. This study offers a timely look into the often-overlooked area, giving insight into the real-world challenges nurses face, and how they succeed in establishing a therapeutic relationship. Six main themes were developed: entering the patient’s world of experience, providing somatic nursing, building relationships, ‘holding the line’, manoeuvring between rules and frameworks and providing good nursing care during coercive treatment. Nursing patients with a severe ED is demanding, requiring both somatic and psychiatric expertise. The nurse must understand the patient's anxiety, rigidity and need of control, and ambivalence. Nursing patients with a severe ED requires clarity and steadiness, and the nurses must cope with resistance. To succeed, teamwork and support from colleagues are needed.

**Supplementary Information:**

The online version contains supplementary material available at 10.1186/s40337-024-01155-0.

## Background

Eating disorders (EDs) are complex mental conditions that can cause extensive suffering for those affected. These disorders can lead to serious somatic complications [1] and have one of the highest mortality rates of mental illnesses [2]. For those worst affected, intensive and long-term follow-up by the health services is usually required. Compulsory treatment is sometimes required when patients are too ill to be able to care for themselves adequately [3].

Several papers were found underlining the importance of a therapeutic alliance between nurse and patient, for treatment to succeed. In a review of research into adolescents with an ED, empathy, supportiveness, willingness to understand and experience of EDs were called for [4]. Studies with nurses also emphasise the importance of a trusting relationship between nurse and patient [5, 6].

The literature clarifies that it can be challenging to establish a therapeutic alliance with patients suffering from an ED. A paper concerning nurses thematised the struggle of developing therapeutic relationships [5]. This corresponds to the findings in a review showing that nurses could be overwhelmed and even angered by patients denying the severity of their somatic situation, opposing the nurse’s core values [6], and of a study investigating how nurses could be overwhelmed by emotions, feelings of incomprehension and being disappointed and frustrated [7]. In Westwood and Kendal`s review, patients recognised tensions between client preferences for psychological interventions and treatments that prioritised physical care. Patients wanted to keep control but felt that nurses tried to control them. Patients stressed that there could be no recovery without their really wanting it. They asked for a holistic approach, considering both physical, psychological and social needs, even if physical needs initially appear most important [4]. Wu and Chen, in a paper studying nurses, found that it can be difficult for patients to alter their mindset [5].

One study investigated the provision of nursing support to patients during mealtimes. Two patterns were categorised as rule adherence and rule bending, describing the challenges for the staff intervening during refeeding [8]. Patients in another study said that inconsistency in treatment could be distressing. They wanted to take an active part at mealtimes, making them more independent in continuing eating properly after discharge [9].

Zugai et al. found that nurses reported that patients, compelled by the psychopathology of Anorexia nervosa (AN), often sought to challenge or undermine their authority. Some nurses experienced this opposition and conflict as demoralising, whereas others were able to maintain confidence in the therapeutic merit of their care. Younger, inexperienced nurses were particularly vulnerable to interactions that diminished their authority, due to their tendency to engage in friend‐like relationships [10]. A study investigated nursing assistants` experiences with manual restraint during feeding. An important finding was that nursing assistants experienced emotional distress, physical exhaustion, and suffered physical aggression and injury [11]. Wright et al., in a study with carers and patients, underlined that therapeutic relationship is important, but that little is known about how this is experienced in adult EDs services. They investigated maternalism and guarding as phenomena occurring within the therapeutic relationships in a specialist EDs unit, and found that, despite the patients being adults, a ‘motherly’ approach was called for [12].

Several papers underline the importance of education, training and support from colleagues. Kudoa et al. report that nursing assistants coped with the distress by talking to colleagues [11]. In another study with nurses, a need for ongoing support for nurses was identified, as extensive nurse and in-service training [5]. One study pointed to collaboration between nurses in the feeding situation as being important. There was a need for strategic seating arrangements, mediating division of labour, the use of verbal and nonverbal communication as collaborative tools and the importance of experiencing a collaborative resource [13]. Daven et al. show, in their study of nurses, the importance of relying on colleagues and routines for building inner security [7].

The aim of our study was to further investigate nurses` views of challenges when nursing adults with a severe ED, and how to succeed in building a therapeutic relationship.

## Research methodology

### Research design

The design of the study was qualitative. Data were collected by conducting individual, qualitative interviews with nurses from a psychiatric hospital ward with adult patients with a severe ED. Our chosen analytical method was Braun and Clarke’s methodological approach to thematic analysis of qualitative research data [14–16].

### Participants and research context

The participants were twelve nurses from an inpatient ward for adult patients with a severe ED (AN or bulimia) at a hospital in Norway. Information about the study was given during a staff meeting, and those wishing to participate contacted the first author. The ward has eight beds, and about 14 nursing positions, plus treatment staff. Most patients are women, between 18 and 25 years of age. A few are in their thirties and forties. Some patients are admitted voluntarily, some compulsorily. The length of stay varies from a few days to several months. The nurses interviewed were two men and ten women between 38 and 70 years old. The participants had between two- and 19 years’ experience from the inpatient wards for adult patients with an ED. All nurses interviewed also had extensive work experience with other patient groups, both within mental and somatic health services.

### Data collection

After obtaining informed consent from the participating nurses, the first author conducted the interviews at the hospital in 2022 using an interview guide (see Supplemental materials 1). The interviews lasted 54 min on average. All interviews were audio recorded and transcribed verbatim. The main research question was: “Can you describe as concretely as possible specific instances from your nursing experience with patients suffering from a severe ED, detailing situations where you provided good nursing”.

### Data analysis

Braun and Clarke’s methodological approach to thematic analysis of qualitative research data was used [14–16]. The analytical approach is inductive, “data-driven” and detached from preconceived theories. The analysis is divided into six phases: familiarising oneself with data, generating initial codes, searching for themes, revising/reviewing themes, defining and naming themes, and finally, producing the report (article). Each author meticulously and iteratively reviewed all the interviews independently and collectively. Relevant passages from each interview were extracted. Text with manifest meaning were extracted and coded, and an initial understanding of possible thematisation developed. By reading across the codes, latent meanings were uncovered, which made it easier to develop themes. The final interpretation of meaning was thus derived from extensive analysis across all the interviews [19]. A COREQ checklist is attached (Supplementary materials 2).

### Ethical considerations

The study was approved by the Norwegian Data protection services for research (Reference no. 947253). All participants provided informed consent, and the data material is anonymised and treated confidentially.

## Results

Six main themes were developed: entering the patient’s world of experience (with the subthemes: understanding the patient’s anxiety, understanding the patient's rigidity and need for control and understanding the patient's ambivalence), providing somatic nursing, building relationships, ‘holding the line’ (with the subtheme: demanding and rewarding), manoeuvring between rules and frameworks and providing good nursing care during coercive treatment.

### Entering the patient’s world of experience

The participants said that an essential aspect of being an ED nurse is trying to understand what the patient is going through. The nurses where both curious and concerned with the patients` ED symptomatology. They stressed the importance of *“entering the patient’s world of experience”* (nurse 6) as a foundation for providing appropriate nursing care.

#### Understanding the patient's anxiety

Several nurses stressed the importance of understanding the ED as an anxiety driven disorder.

*"Never forget the anxiety that underlies ...... Extreme anxiety. Anxiety about losing control, anxiety about disintegrating, anxiety about losing coherence.” (nurse 4).*

This seemed to be a reminder for the nurses to make sure they understood the patient as a basis for providing good care.

*"It's really easy to get tired of watching them sit, they can't eat and rummage around food... but you have to remind yourself that... that you are not sitting there with a child who does not want to eat, you are sitting with a person who has severe anxiety around eating.” (nurse 9).*

One nurse described how a patient was reluctant to accept necessary, and potentially lifesaving, treatment because the anxiety of the disorder was so overwhelming.

*"There was a patient who had such low blood sugar, and then we were going to put gel in her mouth, and then she said I don't know if... I'm more afraid of getting that sugar jelly... I'm more afraid of it than dying of low blood sugar. …I feel goosebumps myself when I think about it.” (nurse 4).*

Several nurses described situations in which they worked with the patient as if they had an anxiety disorder, by exposing them to what they feared.

*"She got a complete breakdown of the food. …And then I just sat down with her and was with her and said, now you must remember to breathe. So, I just sat there. And then she took a little breath and then she recovered and then we calmed it down... "(nurse 7).*

#### Understanding the patient's rigidity and need for control

Being in control of oneself and one’s surroundings was something several nurses described as a central part of the patient’s illness, that was often shown in a rigid, non-flexible and potentially dangerous way.

*"The biggest challenge is that it's a very, very deadlocked ... In other words, it is such an extensive disease, it is so dangerous.” (nurse 3).*

*"There was someone here that had such magical thinking about numbers. She said I can be 38 kg, but nothing more. Then I said, what happens if you get over 38 kg. ‘I'd rather die’." (nurse 1).*

The same nurse described how being in control seemed to be a way of avoiding psychological pain.

*"… it often starts with depression and then they discover starving themselves, then they starve away their feelings and feel better about themselves, and don't have to feel the pain that they have experienced or are experiencing, and gain control over their things or life. Having an ED is very much about being in control of oneself and one's surroundings.” (nurse 1).*

Several nurses stressed the importance of providing a sense of normality as a counterbalance to the patients’ rigid and non-flexible way of being.

#### Understanding the patient's ambivalence

ED nurses understood and described ambivalence as a central part of the ED, shown by many patients being reluctant to commit to therapy and change*. “…where perhaps 'won't' is greater than 'will’.” (nurse 11).*

*"I think that getting treatment, a lot of people who have an ED have a very ambivalent relationship with that. You want to, but then it's so scary to make changes. You become very vulnerable, scared, maybe small..." (nurse 8).*

Some talked about how the ambivalence in the ED poses the risk of nurses taking over the patient’s project, potentially undermining it, with a risk of relapse.

*"Many people who have an ED have an ambivalence towards weight gain. And sometimes it's the staff who kind of do most of the work. But then I think maybe that's how it is at first. …When you end the treatment… you are part of it and …own your own treatment and... Otherwise, it will be our kilos that they gain, and then there is a risk of relapse when they come home." (nurse 8).*

One nurse stressed re-feeding as one important element in helping the patient getting to the point of committing to therapy.

*"…. it's when they connect with treatment and work with and not against. And often they are a little better nourished before they break the code …" (nurse 11).*

Noticing a change in the patient’s ambivalence can occur in several ways. The same nurse described how that changed the whole energy, not only for the patient, but also for the nurse.

*"And when they say I'm going to do this, I'm not going to (just) try anymore, then something happens to the whole energy around, even for me, because it's feedback that now we're starting to... that we are succeeding in what we are doing.” (nurse 11).*

### Providing somatic nursing

The participants said that nursing patients with a severe ED requires solid and holistic nursing competence, knowledge and experience of somatics, as well as mental Illness and health.

*"It's everyday life for those who are hospitalised, the most basic part of everyday life. Food, bed and safe framework to get to sleep and get on their feet, using physical activity in a good way, and that they should not ruin themselves, we are working on.” (nurse 2).*

Several participants stressed the importance of solid somatic competence. A serious ED is a mental disorder that very quickly becomes a somatic condition that can affect all organ systems.

*"You shouldn't have forgotten your somatic, which is fundamental. I use the clinical eye every day." (nurse 4).*

*"You have to observe blood pressure drops, you're going to observe if they get drops in glucose..... They might have heart disease here.... You have to observe if they have oedema." (nurse 5).*

*“They can get renutrition syndrome. You must observe the patient clinically while keeping the blood test results in mind ...” (nurse 6).*

Several statements made by the nurses emphasise that some of these patients have a lot of pain, problems with the stomach and air pain. They also pointed to practical nursing procedures, such as blood tests, medicines and inserting probes that are part of nursing these patients. Some mentioned that nurses must also observe whether patients are getting enough fluids and prevent and treat constipation.

*"They can be so weak that they can hardly get up or move in bed. And there may be pressure sores, or sores on heels. Some patients need an air mattress. So, you really have to think nursing." (nurse 10).*

### Building relationships

The participants emphasised the importance of seeing the whole person, not just the disease, and having a good dialogue with the patient. Creating a ‘we’ so that the patient thinks that it's me and you against the ED. We will do this together.

*"I like to imagine you're like a puzzle in pieces. I don't know who you are, and for me to help you or understand you, we must talk and find out... And to kind of put a puzzle together to get the whole picture." (nurse 8).*

Some mentioned that nurses must be humble. Several of the statements made by nurses emphazise that patients notice who really cares, for example from the handshake, how the nurses are with them, how they ask or talk… about the proximity or the distance: a gut feeling.

One nurse said: *“I think about how I sit, how I have my body. If I'm sitting on an office chair, I always make sure I adjust it down so I'm at the same eye level as the patient." (nurse 4).*

Another said: *"Good care is not knowing best always……And maybe have a lot of knowledge without having to advertise it all the time. … I think good care is meeting them where they are and figuring out and working hard to find out what it is that you understand about yourself and what it is that you want me to help you with." (nurse 3).*

Others reported that they sometimes used situational humour. They said that nurses together could lighten the mood, and draw in the patients, if the mood became too heavy and gloomy. Several talked about how they used various activities as a diversion, to get the focus away from illness and problems, and onto coping and the positive things in life.

*"I could knit, and I could also talk and bake and go for walks and... I have been to the spa with a patient who was going to challenge herself to wear a bikini after she had a new body... Introducing people to a little bit from their own lives. You have to offer yourself a little bit. You use yourself when meeting other people." (nurse 8).*

The participants also agreed that it was important to be professional as a nurse so that one did not take on a role as a friend or parent:

*"I'm not good at being friends with the patient, I don't want to be a friend, I just want to be a nurse, who sees and hears them, I've been very conscious of, that's my role ..... And I'm not their parents. After all, they're grown-ups and there's a point to treating them as adults." (nurse 2).*

Many also talked about how important it is to work well together, and to use each other's strengths, resources and experience, both among nurses, but also in the interdisciplinary collaboration on the ward.

*"The best quality is that we are different. I think that the best quality is that we don't run and copy each other, but that we're unique and ourselves, because that's how it is in life, we're human, we have common traits, but we're different, and they get to practice the reality of life a little bit." (nurse 2).*

### ‘Holding the line’

Several of the participants said that the essence of nursing and care for these patients is to remain steady, to enter the patient's world of experience and take it in, while at the same time knowing and remaining clear that you have a responsibility to lead the patient towards health. There will very often be resistance. Appropriate nursing for these patients implies clarity and being suitably strict.

*"I think the concept of care sometimes gets a little misunderstood, that you attribute it to motherliness ... But that's not it. I think it's about wanting the other to be well. And it's as much about setting boundaries as the opposite. The good, caring framework that also creates a sense of security.” (nurse 9).*

The participants referred to often feeling challenged by these patients. They pointed out that counter transference can easily go unnoticed, leading to common perceptions of the patient spreading readily among the staff group, being highly contagious. They made the point that it is, therefore, important to maintain one’s steadiness and to remain cool in relation to the challenging patient. The nurses stressed that this remaining steady -’holding the line’—in the face of resistance, can be extremely demanding.’

*"We have to see them but be determined. It's very important to be firm and give them frameworks because they're so limitless to escape. ..... There is so much resistance......... Maintaining one’s steadiness when there's so much resistance and you just don't want to and don't realise that if you don't want to, you die." (nurse 7).*

*"You can have the feeling that you're hated, or that you're making it so hard for them. But I find that – and I've received a lot of feedback on this – to stand firm anyway, that you can think that statements that come or situations that arise, or if you shut up or they get angry ..... it's about the disease." (nurse 8).*

Several of the nurses described how, because this’remaining steady’ is so challenging, difficulties can arise in cooperating between colleagues. They pointed to one shift not following up on things decided by an earlier shift, which they described as damaging teamwork.

*"We work as a team, and it's very much about how you are as a person yourself. If you can't resist, and give in, you've destroyed a foundation that's been worked on for an entire shift… And then you must start working again because you meet a patient who says yes, but I was allowed to then... And it isn’t fortunate. Because it doesn't help, it just perpetuates the ED. But, often, it's about personnel not being able to remain steadfast, ... They haven't figured out what they were supposed to do. It's about it being too difficult for some nurses in the team." (nurse 11).*

The same nurse said that perhaps the best nurse was the one whom the patient did not like so much when she was hospitalised, but once the treatment was complete, that patient could say that it was that nurse's ability to’hold the line’ that had been most helpful.

*“I think a patient who has finished treatment and is under her own care and is virtually healthy would say that the best nurse was the nurse who was clear and held on to the framework and dared to ’hold the line’ and do what she needed to do to achieve the goal." (nurse 11).*

#### Demanding and rewarding

The nurses told us that meeting and relating to many of these young patients, who it was often so difficult to help, made an impression on them.

*“It's challenging to think of all these great young people, that they're going to spend so much time on this here, that it's going to be so crucial in their lives…. And that they have such a need to regulate or punish themselves.” (nurse 4).*

They also found it challenging to endure the patient's pain, which could be very exhausting sometimes. The nurses often had to get into heavy and long processes with seriously ill people over time.

*“The worst thing is to see the ones that are not doing well, to see that everything you've done, all the effort, all that information, all the meals... I think I'm up to 4,000 meals... Seeing that it has no effect.” (nurse 3).*

The participants also said that they enjoyed working with these patients, who often took time to get to know, but with whom they often felt that they had a good dialogue when they got to know each other. Several said that they thought ED was an interesting and almost fascinating disease, which challenged the nurses and gave them a sense of mastery. It was also very satisfying when some of these seriously ill patients recovered.

*“When you connect with them well, you connect with them all the way…….I like this group of patients. I enjoy working with them.” (nurse 5).*

*“It's rewarding to be able to help others in that way with such a special and complicated disorder.” (nurse 1).*

### Manoeuvring between rules and frameworks

The rigidity of rules and regulations governing treatment was described. Nurses indicated that there were extensive rules and routines in place to manage treatment effectively. However, they noted that some patients would resist these rigid structures, leading to potential conflicts. The balance between necessary rules and flexibility for individualised care was a topic under consideration.

*“I remember very well, the first years I worked on the inpatient ward, everyone had different meal plans. …We had 12 different meal plans, and there were four of us. It was almost impossible to manage. Mistakes were bound to happen. Therefore, we started leaning towards having meal plans as similar as possible, with minimal individual adjustments, except for allergies and other specific needs. “(nurse 5).*

Nurses also emphasised the distinction between rules and frameworks when discussing compulsory treatment. While recognising the importance of having a treatment framework in place, they expressed concerns about the abundance of rules, which sometimes seemed unnecessary. The challenge lay in creating a distinction between necessary guidelines and rules that hindered adaptability in patient care.

*“There is a difference between rules and frameworks. Because I believe that safe frameworks are necessary. But I'm one of those who think that rules... there are too many of them. Sometimes, we end up contradicting ourselves because we've created a rule based on a specific situation or patient.” (nurse 9).*

One of the nurses mentioned that the patients were often very compulsive, with many rules for themselves, and that all the rules in the ward could help to maintain the patient's self-coercion.

*“These patients also have a lot of rules for themselves, and sometimes the thought crosses my mind that they have infected us a bit as well. There are so many rules here about how things should be. There are a lot of rules here, and I think whether we want it or not, we are also influenced by the patients' world, their universe. … So, we're probably a little bit coloured by that, so maybe sometimes we should try to reduce it a little bit and then face them a little bit more like life is out there, rather than meet their rulesets with our rulesets. I think we can maintain the control and the coercion and the governance with that then. I don't have a good answer as to how we're going to solve it that way, but maybe a little more about room for discretion, a little more room for contextual events.” (nurse 1).*

The need for individualised treatment was highlighted. Nurses acknowledged the importance of having a set of rules but stressed the significance of adapting treatment to suit each patient's unique needs and circumstances. This highlighted the importance of striking a balance between adhering to guidelines and tailoring care to the individual.

### Providing good nursing care during coercive treatment

Patients with life-threatening and severe AN occasionally require nasogastric tube feeding under restraint. In these situations, participating nurses recognised that the best form of care is coercion. At the same time, participants were clear that nasogastric tube feeding under restraint represents the most invasive form of coercion. The nurses were very concerned with safeguarding the patients' autonomy, even though nasogastric tube feeding under restraint was necessary. They also spent a lot of time persuading patients to cooperate and take nourishment to avoid tube feeding [17].

*“First, we ask, then we try [to encourage them to eat]. Oh, we try and try. We try again, and then if it doesn't work, we ask about their thoughts on the matter, if we should insert it [the nasogastric tube]. Typically, two people are involved where one assists... And if the situation doesn't improve, then we must call personnel [from another ward] to come and assist.” (nurse 7).*

Navigating the fine line between being a helper and exercising control over the patient was also described as a dilemma. When nasogastric tube feeding under restraint was performed, this was done in the most discreet manner possible, both for the sake of the patient in question and other patients in the ward. Several participants mentioned situations where they felt that nasogastric tube feeding under restraint was unnecessary and sometimes unethical, and incompatible with good treatment and care. This could particularly be the case in situations where the patients had a body mass index (BMI) that was no longer life-threatening.

*“I feel it's challenging to be a nurse and insert a tube when their BMI is fifteen or maybe sixteen, but the situation around the patient has reached a point where a nutritional decision has been made to ensure they receive nourishment, and they should mostly eat on their own or take the supplement if they find food too difficult ...these with a high BMI, refusing to eat and being tube fed. It's hard! ….” (nurse 2).*

The nurses said they questioned such situations at staff meetings. Some even went so far as to refuse to participate in nasogastric tube feeding under restraint in patients with a high BMI [17].

## Discussion

The aim of the study was to investigate nurses` views of challenges when nursing adults with a severe ED, and how to succeed in building a therapeutic relationship. Six main themes were developed: entering the patient’s world of experience (with the subthemes: understanding the patient’s anxiety, understanding the patient's rigidity and need for control and understanding the patient's ambivalence), providing somatic nursing, building relationships, ‘holding the line’ (with the subtheme: demanding and rewarding), manoeuvring between rules and frameworks and providing good nursing care during coercive treatment.

EDs are complex diseases, which places great demands on the nurse. Somatic nursing and nursing during compulsory treatment are important, but are discussed elsewhere [11, 17–21] and will not be part of the discussion of this article.

An initial essential aspect of ED nursing is trying to understand what the patient is going through. The nurses in this study stressed the importance of understanding the patients’ struggles and of the mechanisms they use to maintain the ED, addressing anxiety, rigidity and control, and ambivalence. At the core of ED psychopathology, we commonly find anxiety regarding food, eating, weight and body shape [22]. Anxiety was mentioned by several nurses as a driving mechanism for the ED, from fear of eating normal types of food, accepting potentially lifesaving treatment, and even, to an extent, fear of losing all sense of themselves. They stressed the importance of challenging the patient’s anxiety through different interventions, for instance breathing techniques and exposure to what they feared. This is supported by Serin and Sanlier, suggesting that nurses need to accept that the patients have fears and help them to strengthen coping mechanisms and techniques to reduce their anxiety level [23]. Patients’ need to be in control, often in a rigid and non-flexible way, was highlighted by the nurses in our study. They gave examples of how stuck patients could seem, in their need of control, to be trying to control not only their fears around food and weight, but also avoiding the psychological pain within, and over, life in general. The literature shows how patients with an ED can show a lack of flexibility [5], high levels of cognitive rigidity [24], and the ego-syntonic nature of ED [25], that can challenge the patient’s ability to commit to treatment. Treatment for EDs can give patients a feeling of loss of control, even a threat to their autonomy [4, 6]. One can understand how anxiety and a feeling of lack of control triggered by treatment interventions and, for some, a fear that health personnel will try to control them, can result in ambivalence in the patient. Ambivalence therefore seems to be a common multifaceted and maintaining mechanism in ED treatment that one cannot overlook in ED nursing. The nurses in this study gave examples of how patients can fear committing to treatment because of ambivalence, to a degree that not wanting to make changes can be greater than wanting to make changes. Ambivalence can result in patients struggling to commit to treatment, and thereby in therapists and nurses having to work very hard to engage the patient with ED. This is in line with previous research on ED and ambivalence [23, 26].

Our results show that nursing patients with a severe ED requires solid and holistic nursing competence, knowledge and experience of somatics, as well as mental Illness and health. The participants stressed the importance of seeing the whole patient, and not just the disease. This requires a holistic approach and good dialogue. The need of a therapeutic alliance is a central finding in other studies [4, 6]. Zugai et al. found that the therapeutic alliance is dependent on nurses’ capacity to maintain their position of power, whilst demonstrating their trustworthiness [27]. Some studies show that nurses experienced developing therapeutic relationships as demanding [5, 7, 28]. The participants in our study were concerned with being professional (nurses), and not adopting the role of friend or parent or mother. As one of them said; *"… the concept of care sometimes gets a little misunderstood, that you attribute it to motherliness … But that's not it. …. it’s about wanting the other well. And it's as much about setting boundaries as it is the opposite. The good, caring framework that also creates a sense of security” (nurse 9).* This contrasts with Wright`s [12] and Zugai et al.`s [10, 27] findings. Wright found that despite the patients being adult, a ‘motherly’ approach was appropriate [12]. This is in line with Zugai et al.`study [27] where nurses and consumers described effective nursing as involving a *‘motherly’* or *‘sisterly’* role adoption. Zugai et al. in another study found that young, inexperienced nurses often tended to engage in friend‐like relationships [10].

One of the main results of our study was “holding the line”. As we have previously seen, the patient's resistance and ambivalence often challenge the nurse's role. The nurse must be able to “hold the line”, tolerate the resistance, tolerate feeling hated. George found that persons with AN can fear that health personnel will try to control them instead of helping them, and as a response, they can become stubborn and critical towards the treatment [28]. Zugai et al. reported that patients with AN often sought to challenge or undermine the nurses’ authority [10]. Westwood and Kendal point out that tensions between what the client wants, and clinical interventions may lead to conflicted treatment processes [4]. Daven et al. found that nurses were sometimes overwhelmed by emotions and disappointed and frustrated [7]. Nurses can be overwhelmed and even made angry by patients denying the severity of their somatic situation, opposing the nurse’s core values [6], and experience carer burden [29]. Working with patients with a severe ED can be a test of patience, and some patients never recover. The participants pointed out that it was important to be clear, unambiguous and suitably strict, maintaining their position of power and holding on to the safe, firm framework. In a study by Hage et al., staff members acknowledged the importance of “rule adherence” related to unit structure and the common established rules for meal completion with medical justification as the main rule [8]. Also, patients stated that nurses contributed significantly to their recovery from AN through structure and responsibility [30]. One participant in our study said that perhaps the best nurse was the one whom the patient did not like so much when she was hospitalised, but once the treatment was complete, that patient could say that it was that nurse's ability to’hold the line’ that had been most helpful.

Patients differ, as do nurses and healthcare personnel, and it is important to have employees with different skills and experience. Many participants in our study said it is crucial to work well together, and to use each other's strengths, resources and experience, among the nurses, but also in the interdisciplinary collaboration on the ward. Nurses are dependent on support from colleagues [7], teamwork [13] and in need of ongoing support [5]. Our results show that nurses are concerned with individual facilitation, and that nursing should not be too rule-driven [8]. Because it feels so demanding to keep “holding the line”, it can sometimes be challenging to cooperate. One consequence may be that the nurses on the next shift do not follow up what has been worked on previously, which is perceived as devastating and demanding in the teamwork. Literature suggests that consistency among the staff is important: if health personnel do not keep to the treatment plan, regardless of ambivalence from the patient, the treatment could fail [28]. A certain amount of inconsistency within treatment implementation, may cause distress for some patients [9]. One of the nurses put it this way: *"We work as a team, but it's very much about how you are as a person yourself. If you can't resist and give in, you've destroyed a foundation that's been worked on for an entire shift… And then you must start working again because you meet a patient who says yes, but I was allowed to then… And that's nothing fortunate. Because it doesn't help, it just perpetuates the ED…. " (nurse 11).* This nurse pointed out that not only the individual nurse, but the whole team must work together and "hold the line" collectively. Ultimately, the nurses suggested providing a sense of teamwork as an essential element in challenging the patients` ambivalence, arguing that ‘*it is we and you against the ED’.*

### Limitations and strengths

The participants in this study were twelve nurses from one ward, at one hospital, and consisted mainly of experienced nurses, ten women and two men. All who consented to participate were interviewed, and when the last interviews were analysed, no new issues emerged, indicating that saturation of the data material had been achieved. More participants from other hospitals, including less experienced nurses, and more male nurses, could have provided more nuance in the findings. The authors consist of one nurse without clinical experience with patients with an ED, but extensive experience as a researcher in the field, one psychologist and one psychiatrist, both with solid experience in the field. It is original, and it can be seen as a strength that the role of the ED nurse has been analysed and discussed by other experienced clinicians. One psychiatric nurse with solid clinical experience with patients with EDs has read and commented on the manuscript. This can be considered a strength. Also, this study offers a look into the often-overlooked area of nursing care for patients with a severe ED, giving insight into the real-world challenges nurses face, from nurses` own perspectives.

## Conclusion

The aim of the study was to investigate nurses` views of challenges when nursing adults with a severe EDs, and how to succeed in establishing a therapeutic relationship. Six main themes were developed: entering the patient’s world of experience (with the subthemes: understanding the patient’s anxiety, understanding the patient's rigidity and need for control and understanding the patient's ambivalence), providing somatic nursing, building relationships, ‘holding the line’ (with the subtheme: demanding and rewarding), manoeuvring between rules and frameworks and providing good nursing care during coercive treatment. Nursing care for patients with a severe ED is demanding, requiring both somatic and psychiatric expertise. The nurse must understand the patient's anxiety, rigidity and need for control, and ambivalence. Nursing patients with a severe ED requires clarity, and steadiness and the nurses must cope with resistance. To succeed, teamwork and support from colleagues are needed.

### Implications for practice and further research

Serious EDs are psychiatric disorders with potentially life-threatening consequences, and it is important to develop knowledge about nursing for this patient group. Future research could include nurses from more hospitals, more male nurses and junior nurses. It would also be interesting to interview patients and their families about what they consider appropriate nursing care for patients with a severe ED.

## Supplementary Information

Supplementary Material 1

Supplementary Material 2

## Data Availability

The datasets associated with this study are not publicly available as consent to publish full datasets was not sought and obtained from participants.

## Abbreviations

AN: Anorexia nervosa
BMI: Body mass index
EDs: Eating disorders
