# Supplementary material for: “Holding the line”—relationship building and challenges when nursing adults with a severe eating disorder
Source: J Eat Disord. 2024 Dec 2;12:198. doi: 10.1186/s40337-024-01155-0 (PMC11610163; doi:10.1186/s40337-024-01155-0)
Supplement: Supplementary file 2 — Supplementary Material 2 [file 40337_2024_1155_MOESM2_ESM.docx]

**Interview guide:** (individual interview: nurses with experience of adult patients with a severe ED)

How old are you?

What training do you have?

What work experience do you have?

How long have you been working with patients with eating disorders?

Tell us about your workplace

Share some experiences that have made an impression on you as an eating disorder nurse

Tell about situations where you felt that you were providing good nursing care – preferably as concrete as possible

Tell us about a situation where you experienced an ethical dilemma

What are your thoughts on rules and discretion?

What are your thoughts on the use of coercion?

How have you experienced working with relatives?

What is particularly rewarding about working with this patient group?

What is particularly difficult/challenging about working with this patient group?

Why does this group of patients need nursing?

What do you think the patient/next of kin think is good nursing?

Do you have anything else important to convey that others can learn something from?

What would you ask if you were the scientist?

I have now asked you as a nurse what you think is good nursing/ care for people with

eating disorders. What do you think a patient/family member would answer the same question?

Is there anything important we haven't talked about?
